# Development of a small animal model to simulate clinical stereotactic body radiotherapy-induced central and peripheral lung injuries

**DOI:** 10.1093/jrr/rrt234

**Published:** 2014-02-20

**Authors:** Zhen-Yu Hong, Sung Ho Eun, Kwangwoo Park, Won Hoon Choi, Jung Il Lee, Eun-Jung Lee, Ji Min Lee, Michael D. Story, Jaeho Cho

**Affiliations:** 1 Department of Radiation Oncology, Yonsei University College of Medicine, 50 Yonsei-ro, Seodaemun-gu, Seoul, 120-752, South Korea; 2 Department of Radiation Oncology, University of Texas Southwestern Medical Center, Dallas, Texas, USA

**Keywords:** SBRT, animal model, pneumonitis, fibrosis

## Abstract

Given the tremendous potential of stereotactic body radiotherapy (SBRT), investigations of the underlying radiobiology associated with SBRT-induced normal tissue injury are of paramount importance. This study was designed to develop an animal model that simulates centrally and peripherally located clinical SBRT-induced lung injuries. A 90-Gy irradiation dose was focally delivered to the central and peripheral areas of the left mouse lung with an image-guided small-animal irradiation system. At 1, 2 and 4 weeks after irradiation, micro-computed tomography (micro-CT) images of the lung were taken. Lung function measurements were performed with the Flexivent^®^ system (SCIREQ^©^, Montreal, Canada). For the histopathological analysis, the lungs were fixed by perfusing with formalin, and paraffin sections were stained with hematoxylin and eosin and Masson's Trichrome. Gross inspection clearly indicated local lung injury confined to the central and peripheral areas of the left lung. Typical histopathological alterations corresponding to clinical manifestations were observed. The micro-CT analysis results appeared to correlate with the histopathological findings. Mouse lung tissue damping increased dramatically at central settings, compared with that at the control or peripheral settings. An animal model to simulate clinical SBRT-induced central and peripheral lung injuries was developed and validated with histopathological, radiological and functional analyses. This model increases our understanding of SBRT-induced central and peripheral lung injuries and will help to improve radiation therapy in the future.

## INTRODUCTION

For patients with medically inoperable, early-stage non-small-cell lung cancer (NSCLC), stereotactic body radiotherapy (SBRT) has emerged as a promising surrogate to conventional fractionated radiotherapy [1, 2]. This extreme hypofractionation, however, is a significant break from prior radiobiological understandings and experiences of tissue toxicity.

Radiation pneumonitis and fibrosis can arise in the lung after the irradiation of malignant thoracic disease. These complications can range from relatively mild to life threatening, depending on a number of factors, including the total radiation dose, fractionation schedule, lung irradiation volume, and irradiated area location [3]. There is little existing experience with SBRT for central lung tumors because these tumors are relatively rare. In addition, common SBRT dosing schedules, such as three fractions of 20 Gy, are not safe because of the proximity of the trachea, main stem bronchus, esophagus, and heart. Serious complications have been reported, including death consequent to bacterial pneumonia, pericardial effusion, radiation pneumonitis, or massive hemoptysis [4, 5]. By increasing the number of fractions and reducing the fractional doses, some groups have reported the successful treatment of central lung tumors with minimal complications [6]. However, others have reported Grade 5 toxicity related to stereotactic radiotherapy treatment [4, 7–9]. Guidelines related to lung SBRT toxicity are based on extremely limited clinical data, and most have not been validated. Under these circumstances, an animal model that simulates clinical SBRT-induced central and peripheral lung injuries could be helpful in the provision of valuable guidelines for clinical and molecular biological studies.

In a previous study [10], we established an experimental model and image-guided animal irradiation system to study high dose-per-fraction irradiation such as SBRT at volumes analogous to those used in human beings. Furthermore, we found that ablative irradiation could induce obvious fibrosis at 8 weeks after irradiation, which is usually considered a late effect. Because we examined pathological changes at only one timepoint in that study, we do not know what happened at the earlier timepoints. Moreover, the location effects on lung pathology and function were not investigated.

In this study, by taking advantage of this image-guided animal irradiation system, we investigated ablative-dose focal irradiation-induced lung injuries at two different locations to simulate SBRT-induced central and peripheral lung injuries at several earlier timepoints. The ultimate goal of this study was to develop an understanding of the central radiobiological parameters of the biological foundation of the potent hypofractionation used in stereotactic radiosurgery (SRS) and SBRT so that these therapies can be optimized for greater tumor control and limited adverse normal tissue responses.

## MATERIALS AND METHODS

### Irradiation system

**Fig. 1.**
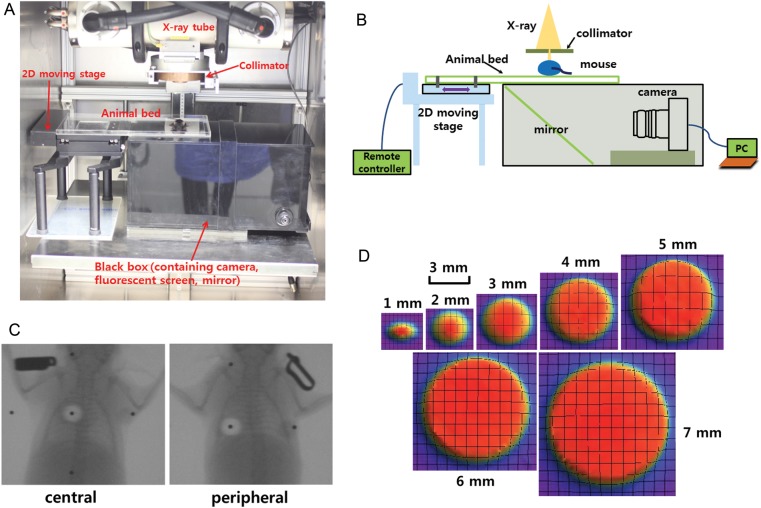
Establishment of an image-guided focal irradiation system. (**A**) An image-guided focal irradiation system was established at Yonsei University. (**B**) Schematic diagram of the irradiation system. (**C**) Image-guided localization to the central and peripheral fields of the mouse left lung. A 90-Gy dose was given to the mouse left lung in a single fraction. A 3-mm collimator was used to produce focal irradiation beams. (**D**) Sharp and steep dose distributions of each collimator at a 1-cm depth, from which the full width half maximum (FWHM) was determined to fit the mouse irradiation size.

**Fig. 2.**
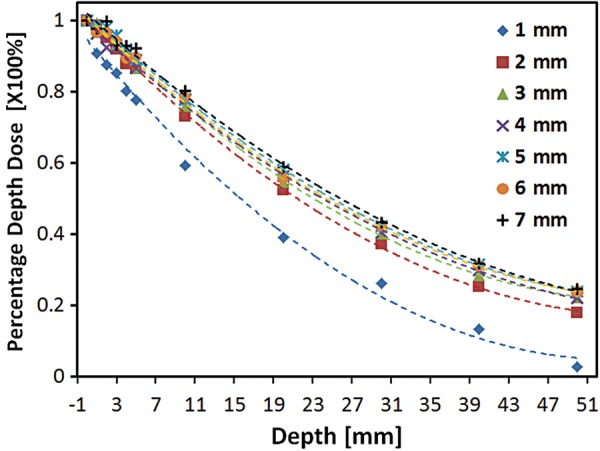
Depth–dose relationships with different collimator sizes. The collimators generated a cone beam that ranged from 1–7 mm in diameter. The percentage depth doses (PDDs) were measured with GAFCHROMIC EBT2 film.

Radiation was delivered with an X-RAD 320 (Precision, North Branford, CT, USA), equipped with a collimator system composed of 5-cm-thick copper to produce focal radiation beams. The image-guided device comprised a motorized, 2D moving stage for accurate positioning, a fluorescent screen (Kodak Min-R 2000; Carestream Health Inc., Rochester, NY, USA), and a charge-coupled device (CCD) camera (650D; Canon Inc., Tokyo, Japan) controlled by a mobile computer (Fig. 1A and B). The collimators generate a cone beam that ranges from 1–7 mm in diameter. The percentage depth doses (PDDs) were measured with GAFCHROMIC EBT2 film, as shown in Fig. 2. The aluminum filtered X-ray beam dose rate was 19.7 cGy/s, measured at 320 kV and 12.5 mA by using a cylindrical ionization chamber within a solid water phantom (PTW, RW3) at a 2-cm depth and a 17-cm source-to-surface distance (SSD). The output was calibrated as recommended by the American Association of Physicists in Medicine TG-61 report [11]. Furthermore, the dose distribution at a depth of 1 cm was considered for the proper collimator size. Figure 1D shows the sharp and steep dose distributions of each collimator, from which we could determine the full width half maximum (FWHM) to fit the mouse irradiation size.

### Mouse irradiation

All studies involving mice were approved by the Yonsei University Medical School Animal Care and Use Committee. Five adult (10-week-old) male C57BL/6 mice were housed per cage and were allowed to acclimate for 1 week after shipping before treatment. To mimic SBRT conditions by irradiating only a small volume, we selected a 3-mm collimator to administer a 90-Gy dose to the left lung. Image guidance was used to administer the 3-mm diameter irradiation field to the left main bronchus, which served as the counterpart of the clinical central area. The peripheral setting was located as far as possible from the central setting to serve as the counterpart of the clinical peripheral area (Fig. 1C). During irradiation, the mice were anesthetized with an intraperitoneally administered mixture of 30 mg/kg of zoletil and 10 mg/kg of rompun. The four legs of the mice were additionally fixed with adhesive tape. At 1, 2 and 4 weeks after irradiation, the mice were sacrificed. Three mice were allocated per group, and the experiment was repeated twice.

### Fixation

On the appropriate day after irradiation, the mice were anesthetized. The lungs were slowly inflated via tracheal perfusion with phosphate buffered 4% formalin, using an 18-gauge needle attached to a syringe. The lungs were immersed in fixation solution for several days until they were completely fixed, after which they were photographed on a black background.

### Histopathology and immunohistochemistry

To visualize histopathological damage in sham-irradiated and irradiated tissues at each predetermined timepoint, hematoxylin and eosin (H&E) and Masson's Trichrome staining were performed as previously described [10]. Alveolar inflammation was scored as previously described [12] on a scale of 0–5, with 0 indicating no alveolar inflammation and 5 indicating complete tissue consolidation. Semi-quantitative assessments of the degree of interstitial fibrosis were assessed by using a predetermined numerical scale of 0–8, based on the Ashcroft scoring method [13]. The criteria for this scoring were based on histological features such as the alveolar wall thickness, fibrotic damage to the lung structures, and fibrous lesions.

### Lung functional assessment

Lung function in irradiated mice was evaluated with the Flexivent system (Flexivent^®^; SCIREQ^©^, Montreal, QC, Canada), which measures flow–volume relationships in the respiratory system. This system uses forced oscillation to discriminate between airway and lung tissue variables. The associated protocols adhered to the manufacturer's instructions. Briefly, after anesthetization, mice were connected to a computer-controlled small-animal ventilator and quasi-sinusoidally ventilated with a tidal volume of 10 ml/kg at a frequency of 150 breaths/minute. As spontaneous breathing should be avoided with this technique, mouse breathing was stabilized with an automatic ventilator until the interruption wave disappeared. All perturbations were performed sequentially until three acceptable measurements (coefficient of determination [COD] > 0.95) were recorded for each subject, from which an average was calculated. The lung tissue variables of inspiratory capacity, tissue damping, and hysteresis were measured.

### Computed tomography scanning

Computed tomography (CT) images were collected on a volumetric CT scanner (NFR-Polaris-G90MVC; NanoFocusRay, Iksan, Korea) at 50 kVp, 180 µA and 150 mGy. Images were acquired at 142 ms per frame and 700 views and were reconstructed by using the volumetric cone-beam reconstruction (FDK) in-line/off line mode. The reconstructed image was 1232 × 1120 pixels and contained 512 slices. To generate 3D images, the final reconstructed data were converted to the Digital Imaging and Communications in Medicine (DICOM) format by 3D-rendering software (Lucion; MeviSYS, Seoul, Korea). Voxels thresholds of −700 and −350 Hounsfield units (HU) were required to obtain reasonable 3D images that defined the lung surface. Volumetric analysis was performed from 3D lung images created through isosurface profiling. Lung parenchyma density measurements were obtained from the average HU within a circle (3.0-mm diameter) in the irradiated field that avoided both the high-density large blood vessels and motion artifacts at the lung boundary.

### Statistics

Data were statistically analyzed with the Student's *t*-test, and differences with a *P*-value <0.05 were considered significant.

## RESULTS

### Gross morphology

**Fig. 3.**
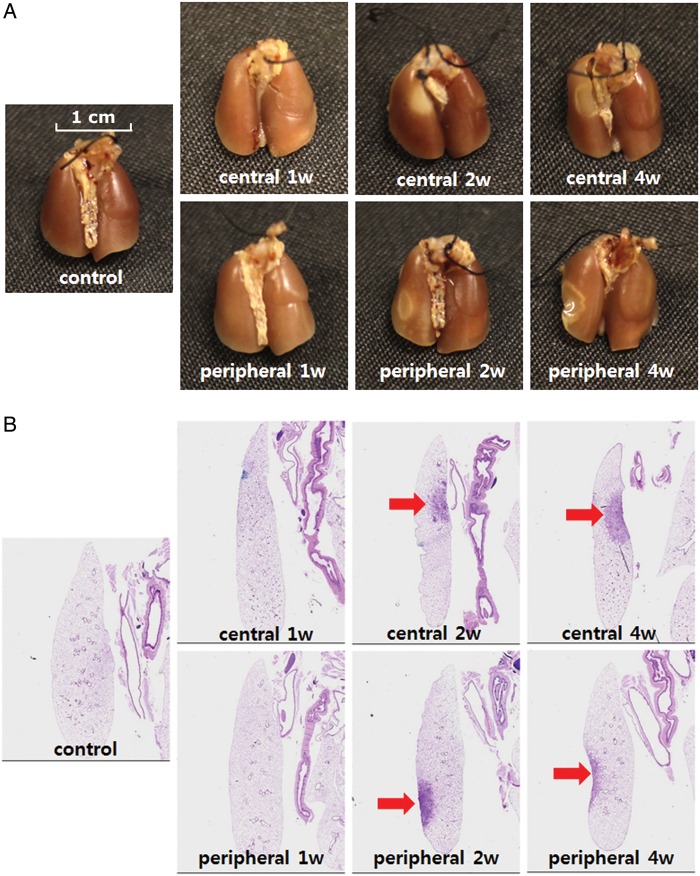
Morphologic observation. (**A**) Representative gross findings. Mice were sacrificed at the indicated timepoints after irradiation, 4% buffered paraformaldehyde in phosphate-buffered saline (PBS) was instilled via the trachea, and the lungs were immersed in fixation solution for several days. Lungs were photographed after complete fixation. (**B**) Hematoxylin–eosin-stained lung sections from a minimum of three mice were examined at each timepoint. Representative images of the major findings are shown. The arrows indicate the injury area (magnification: ×12.5).

We observed gross lung morphology at each timepoint after focally delivering doses of 90 Gy to the central and peripheral areas of the mouse left lung (Fig. 3A). At 1 week after irradiation, we did not observe morphological abnormalities on the central or peripheral lung surfaces, compared with the control. Two weeks after irradiation, inflammation, characterized by a dark area with a white ring-like boundary, appeared in the peripheral irradiated area. In the central irradiated area, however, we observed a white oval-shaped injury. Four weeks after irradiation, the healing process was evident, characterized by tissue contraction, fibrous scar tissue, and a clear boundary between the injured and normal tissue. Overall, the gross inspection revealed a clear local lung injury confined to the central and peripheral areas of the left lung.

### Histopathological damage

**Fig. 4.**
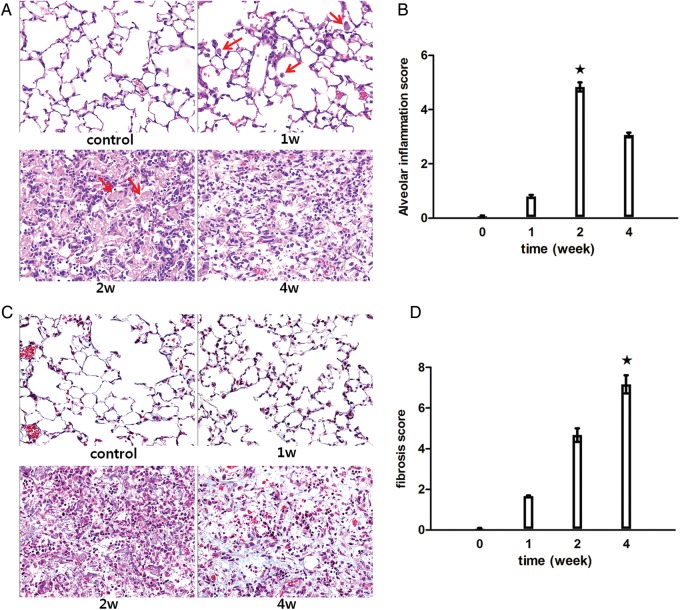
Histopathologic analysis. (**A**) Hematoxylin–eosin-stained lung sections from a minimum of three mice were examined at each timepoint. Representative images of the major findings are shown. The arrows indicate the inflammatory cells present at Week 1 and hyaline material present at Week 2 (magnification: ×400). (**B**) Alveolar inflammation was scored on a scale of 0–5, with 0 indicating no alveolar inflammation and 5 indicating complete tissue consolidation (filled stars, *P* < 0.05 *vs* any other group). (**C**) Lung sections were stained with Masson's Trichrome stain to visualize collagen deposition. Representative micrographs (magnification: ×400) of stained lung tissue are shown at each timepoint. (**D**) Semi-quantitative assessments of the degree of interstitial fibrosis were determined by using a predetermined numerical scale of 0–8, based on the Ashcroft scoring method (filled stars, *P* < 0.05 *vs* any other group).

Significant abnormalities consequent to focused ablative dose irradiation were observed in the H&E-stained sections collected at different timepoints (Figs 3B and 4A). The sequential and pathological alterations observed in the central and peripheral locations demonstrated synchronous changes. At 1 week after irradiation, mild interstitial inflammatory cell infiltration was observed. Two weeks after irradiation, intra-alveolar hyaline material was observed. Regarding focal irradiation, numerous foamy macrophages aggregated in a distal part of the irradiated area, whereas hemosiderin-laden macrophages were observed in the center of the irradiated area. Four weeks after irradiation, the hyaline materials fragmented and disappeared to a certain extent, and fibrous exudates were present in the air spaces along with inflammatory cell infiltration. The alveolar inflammation score at 2 weeks post-irradiation was significantly higher (4.8 ± 0.16), compared with that in the other two groups (*P* < 0.05, Fig. 4B).

To further confirm fibrosis, lung sections were stained with Masson's Trichrome to visualize collagen deposition. Representative micrographs of the stained lung sections are shown in Fig. 4C. No collagen was detected at 1 week after irradiation. At 2 weeks after irradiation, small amounts of collagen were detected in the intra-alveolar and interstitial areas. At 4 weeks after irradiation, extensive collagen was observed, correlating with late-stage fibrosis. The lung fibrosis score at 4 weeks post-irradiation was significantly higher (7.16 ± 0.44), compared with that in the other two groups (*P* < 0.05, Fig. 4D).

### Micro-CT analysis

**Fig. 5.**
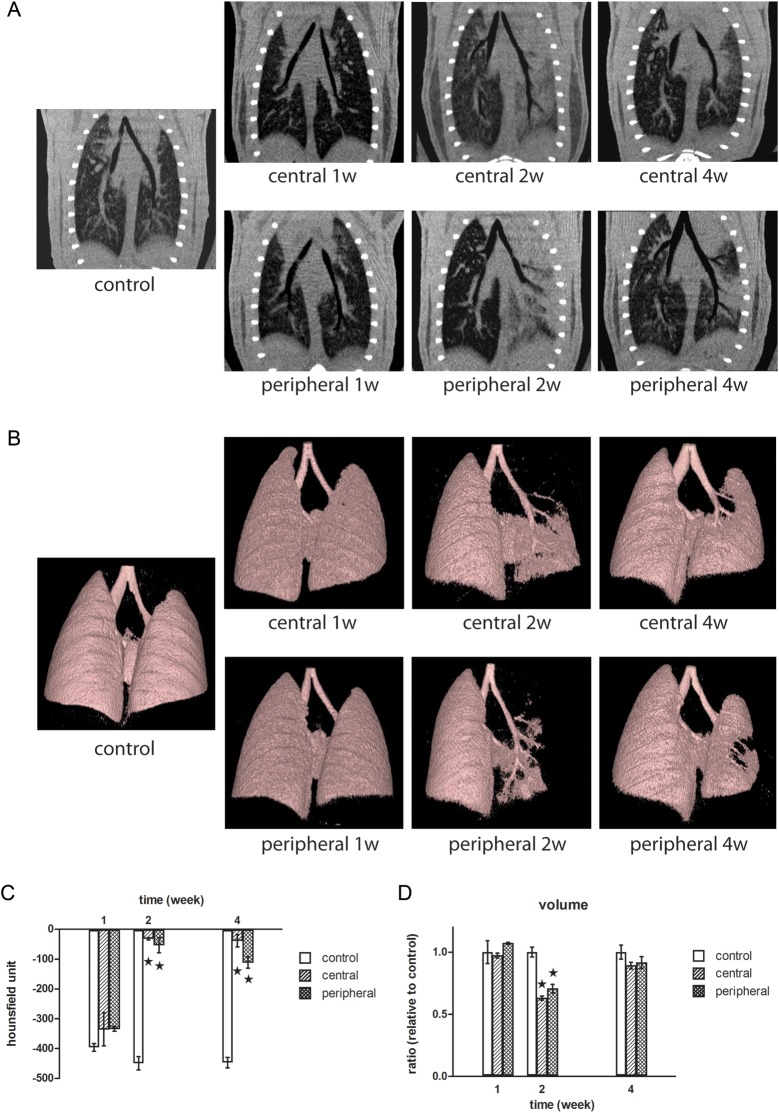
Micro-CT analysis. (**A**) Typical micro-CT images of coronal sections cutting through the main bronchus at the indicated timepoint are shown. (**B**) Areas of density between −700 and −350 HU on 3D micro-CT images are shown in pink. (**C**) Average HU in the irradiated area (filled stars, *P* < 0.05 vs control). (**D**) Volumetric analysis was performed from 3D images of the lung that were created through isosurface profiling (filled stars, *P* < 0.05 vs control).

Micro-CT, which is CT conducted on a microscopic level, is comparable with clinical CT in human subjects [14]. Micro-CT coronal sections taken through the main bronchus are shown in Fig. 5A. One week after irradiation, there were no obvious changes in the central and peripheral irradiated fields. Two weeks after irradiation, ground-glass opacities could be observed throughout the left lung in both the central and peripheral irradiated fields. Four weeks after irradiation, sharp margin consolidation that was confined to the central and peripheral irradiated fields was observed. In the central setting, the administration of 90 Gy of focal irradiation to the left main bronchus did not obstruct the airway at any timepoint. The 3D images in Fig. 5B provide a more intuitive indication of the airway obliteration than do the 2D coronal sections and support the similar findings. The lung densities in the irradiated areas were evaluated as averaged Hounsfield units. As shown in Fig. 5C, the lung densities in both the central and peripheral settings increased significantly at 2 and 4 weeks, compared with the control. The two settings, however, did not differ significantly from each other. Two weeks after irradiation, although the CT-measured whole lung volumes in the central and peripheral settings were significantly lower than that of the control, the two settings did not differ significantly from each other (Fig. 5D). Four weeks after irradiation, the whole lung volume recovered to near-control values. Overall, the results of the micro-CT analysis appeared to correlate with the histopathological findings.

### Functional study of irradiation-induced lung injury

**Fig. 6.**
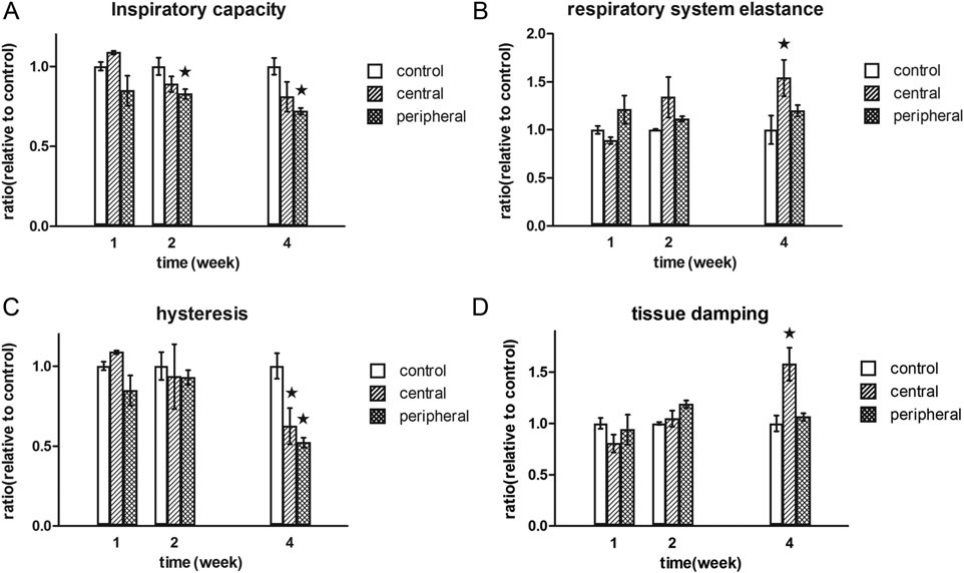
Functional evaluation of mouse lung after irradiation. At the indicated timepoints after irradiation, functional measurements of the mouse lung were collected with a flexivent system. (**A**) Inspiratory capacity (filled stars, *P* < 0.05 vs control), (**B**) respiratory system elastance (filled star, *P* < 0.05 vs control), (**C**) hysteresis (filled stars, *P* < 0.05 vs control), and (**D**) tissue damping (filled star, *P* < 0.05, central vs control and peripheral).

Changes in lung function were evaluated by measuring the forced-oscillation lung mechanics (Fig. 6). The inspiratory capacity decreased significantly in mice with peripheral area irradiation, compared with controls (*P* < 0.05) at 2 and 4 weeks after irradiation (Fig. 6A). Respiratory system elastance increased significantly in mice with central area irradiation, compared with controls, at 4 weeks after irradiation (*P* < 0.05; Fig. 6B). Hysteresis, which reflects pulmonary recruitability, decreased in both the central and peripheral areas at 4 weeks after irradiation (Fig. 6C). Tissue damping, which reflects the energy dissipation in lung tissues, increased dramatically in the central areas, compared with the control and peripheral areas, at 4 weeks after irradiation (*P* < 0.05; Fig. 6D). Overall, the lung mechanics measured by the forced-oscillation method in our animal model generally displayed signs of respiratory distress at 4 weeks after irradiation, compared with the mechanics in control mice. Furthermore, respiratory system elastance and tissue damping are potential parameters that could be used to distinguish functional differences between central and peripheral irradiation.

## DISCUSSION

This preliminary and exploratory study was designed to develop an animal model that would simulate clinical SBRT-induced lung injuries in central and peripheral locations. A previously established, image-guided animal irradiation system was used to administer ablative doses of focal irradiation to different lung locations. At several timepoints after irradiation, pathological, radiological and functional studies were performed to verify toxicities.

Experiments in a conventional fractionated radiotherapy (CFRT) mouse model, which utilizes low-dose whole or half-lung irradiation, generally result in diffuse radiation-induced lung injuries (RILIs) [15–19]. In our system, however, RILI was confined to a focal irradiated area.

Not only did the gross findings of our study differ from those of previous CFRT simulating models, but the histopathological findings also revealed some interesting differences. Fibrin-rich serum proteins are usually discovered after radiotherapy, and in some patients these are associated with hyaline material formation [20, 21]. While the typical hyaline materials characteristic of human acute radiation pneumonitis do not occur in most other mammals (including rats and rabbits) [22], they are occasionally observed in a few strains of mice, including C57BL/6 mice treated with low-dose irradiation [23]. Consistent with the results of a previous study, we observed hyaline materials in the irradiated field in C57BL/6 mice treated with ablative-dose irradiation. Nonetheless, the emergence time differed from that of the CFRT mouse model. Some research groups report several months are required to generate hyaline materials after CFRT with an irradiation dose range of 2–32 Gy [24–27]. In our study, the hyaline materials appeared at only 2 weeks after irradiation. The emergence time of the hyaline membrane seems to be related to the radiation dose. Regarding fibrosis, it is clear that the response to 5 Gy was negligible, and after 10 Gy, the initial increase in collagen was not progressive during the 9-month experimental period [28]. In contrast, overt fibrosis was observed only at 4 weeks after irradiation in our system. One of the most striking findings was that extensive inflammatory cell infiltration and a slight collagen deposition were simultaneously observed, suggesting an overlap of pneumonitis and the fibrotic phase. CFRT is well known to induce dissociation between the two distinct types of lung damage, pneumonitis and fibrosis, which occur at 3–6 months and 6 months after radiation, respectively, in both animals and humans [29, 30].

Because gross morphology was evaluated after inflating the mouse lungs with a fixative solution, we were not able to investigate *in vivo* malfunctions, such as obstructions, that usually occur in injured lungs. These pathologies, however, can easily be investigated by micro-CT analysis. In the micro-CT images, we clearly saw a loss of air space during the pneumonitis stage that dramatically recovered during the late stage of fibrosis (Fig. 5B). Moreover, typical micro-CT manifestations of SBRT-induced lung injuries, such as ground glass opacity and consolidation [31], were detected at 2 and 4 weeks after irradiation (Fig. 5A). Volumetric measurements can also be used to monitor the course of disease (Fig. 5D). Overall, the micro-CT results in our study demonstrated that this assay could be used to detect and quantify ablative and focally delivered radiation-induced lung injuries.

In addition to the histopathological and micro-CT studies, the functional dependencies and influences of the organized tissues must be assessed through meticulous studies of physiological changes. Recent reports have strongly advocated the re-evaluation of traditional histological and biochemical tools with which to quantify pulmonary injuries, as human trials of pulmonary fibrosis mostly use lung function parameters [32, 33]. Hysteresis is an important phenomenon that is readily seen in pressure–volume (P–V) curves; the larger the hysteresis, the higher the lung recruitability. In patients with acute respiratory distress syndrome (ARDS), hysteresis decreased as the positive end-expiratory pressure (PEEP) increased because less of the lung had collapsed at the beginning of the P–V curve [34]. In our study, a decrease in lung hysteresis after 4 weeks can be interpreted to mean that an ablative dose of focal irradiation significantly reduced lung collapse, possibly due to the enhanced fibrotic tension.

There have been concerns that patients with central lung lesions are at an increased risk of high-grade toxicity. The 4-year results of a phase II trial at the University of Indiana showed that SBRT for lung tumors resulted in an almost 3-fold increase in Grade 3–5 toxicity in patients with central versus peripheral tumors (27.3% vs 10.4%, *P* = 0.088) [5]. Although this difference was not statistically significant, the data have raised concerns among clinicians. In addition, Song *et al.* [35] reported on nine patients with central tumors who were treated with SBRT (40–60 Gy in three or four fractions), three (33%) of whom developed Grade 3–5 pulmonary toxicity. In our functional study, the respiratory system elastance and tissue damping results suggest a mechanical difference between central and peripheral settings. Tissue damping is closely related to tissue elastance and reflects energy dissipation in the lung tissue. Although the ablative focally delivered radiation cannot induce stenosis in the main bronchus, which is visible by micro-CT, it affects the respiratory mechanical energy dissipation to a certain extent. Tissue damping or energy dissipation is closely related to tissue resistance and reflects either changes in the physical properties of the tissue or heterogeneity in the regional airways [36]. Fibrotic tissue within the central area would induce traction to the main bronchus, resulting in low mobility or stiffness of the main bronchus. Moreover, radiation might induce significant damage to the visco-elastic features of the main bronchus. These changes in the main bronchus would induce a significant alteration of the airflow mechanics through the bronchus to the entire left lung, which would manifest as significant increases in tissue damping (energy dissipation) in central settings. Nonetheless, the existence of fibrosis in the peripheral field only influences a local limited area of the lung volume, which might explain why minor alterations in tissue damping were detected. Hence, lung functional measurements might serve as a valuable adjunct with which to distinguish between central and peripheral pulmonary injuries. Based on our results, mechanical lung function measurements at later timepoints, when fibrotic remodeling has had sufficient time to develop, are sensitive and reliable surrogates of lung injuries at different locations after ablative dose focal irradiation. On one hand, these parameters reflect whole-lung visco-elastic behavior and could serve as novel endpoints of lung fibrosis. On the other hand, a functional experiment that compares focal RILI consequent to the delivery of an ablative dose with large RILI consequent to the delivery of a conventional fractionated low dose in this model is needed in future studies.

One of the limitations of our study is that we investigated the RILI in normal, tumor-free tissues. In a tumor environment, direct interactions between the tumor cells and normal cells, or indirect interactions mediated by the cytokines secreted by these cells, might influence the RILI to some extent. Although these factors were ignored, we believe that our findings remain significant with regard to evaluations of RILI in normal tissue caused by focally delivered ablative radiation.

An animal model that simulates clinical SBRT-induced lung injuries in the central and peripheral lung was developed and validated with histopathological, radiological and functional analyses. This model increases our understanding of SBRT-induced central and peripheral lung injuries and could help to improve radiation therapy in the future.

## FUNDING

This work was supported by the Nuclear Research and Development Program (Grant No. 2011-0031695) and the Radiation Technology R&D program (Grant No. 2013042978) through the National Research Foundation of Korea, funded by the Ministry of Science, ICT, and Future Planning, and by a faculty research grant from the Yonsei University College of Medicine for 2010 (6-2010-0062).
